# Ultrasonic vocalization in rats self-administering heroin and cocaine in different settings: evidence of substance-specific interactions between drug and setting

**DOI:** 10.1007/s00213-016-4247-4

**Published:** 2016-03-10

**Authors:** Riccardo Avvisati, Laura Contu, Emiliana Stendardo, Caterina Michetti, Christian Montanari, Maria Luisa Scattoni, Aldo Badiani

**Affiliations:** Department of Physiology and Pharmacology “Vittorio Erspamer”, Sapienza University of Rome, Rome, Italy; Istituto Superiore di Sanità, Rome, Italy; Sussex Addiction Research and Intervention Centre (SARIC), School of Psychology, University of Sussex, Brighton, UK

**Keywords:** Ultrasonic vocalizations, USVs, Drug abuse, Cocaine, Heroin, Self-administration, Emotion, Environment, Context, Setting, Reward, Affect

## Abstract

**Rationale:**

Clinical and preclinical evidence indicates that the setting of drug use affects drug reward in a substance-specific manner. Heroin and cocaine co-abusers, for example, indicated distinct settings for the two drugs: heroin being used preferentially at home and cocaine preferentially outside the home. Similar results were obtained in rats that were given the opportunity to self-administer intravenously both heroin and cocaine.

**Objectives:**

The goal of the present study was to investigate the possibility that the positive affective state induced by cocaine is enhanced when the drug is taken at home relative to a non-home environment, and vice versa for heroin.

**Methods:**

To test this hypothesis, we trained male rats to self-administer both heroin and cocaine on alternate days and simultaneously recorded the emission of ultrasonic vocalizations (USVs), as it has been reported that rats emit 50-kHz USVs when exposed to rewarding stimuli, suggesting that these USVs reflect positive affective states.

**Results:**

We found that Non-Resident rats emitted more 50-kHz USVs when they self-administered cocaine than when self-administered heroin whereas Resident rats emitted more 50-kHz USVs when self-administering heroin than when self-administering cocaine. Differences in USVs in Non-Resident rats were more pronounced during the first self-administration (SA) session, when the SA chambers were completely novel to them. In contrast, the differences in USVs in Resident rats were more pronounced during the last SA sessions.

**Conclusion:**

These findings indicate that the setting of drug taking exerts a substance-specific influence on the ability of drugs to induce positive affective states.

## Introduction

Previous experiments have shown that the setting of drug taking exerts a powerful influence on the rewarding effects of heroin and cocaine and that this influence is substance-specific. Cocaine self-administration (SA), for example, is greatly facilitated when rats self-administer the drug in an environment that is distinct from the home environment (Non-Resident rats) relative to rats for whom the SA chamber is also the home environment (Resident rats) (Caprioli et al. 2007a, b). Non-Resident rats also exhibit greater motivation for cocaine SA than Resident rats, as indicated by progressive ratio reinforcement schedule procedures. In contrast, Resident rats self-administer more heroin than Non-Resident rats and also exhibit greater motivation in break-point procedures (Caprioli et al. 2008). Furthermore, Non-Resident rats tend to prefer cocaine to heroin in a choice procedure, whereas Resident rats tend to prefer heroin to cocaine (Caprioli et al. 2009). Finally, Non-Resident rats are more vulnerable to relapse into cocaine seeking (in response to cocaine primings administered after a period of extinction) than Resident rats, whereas Resident rats are more vulnerable to relapse into heroin seeking than Non-Resident rats (Montanari et al. 2015). These modulatory effects of setting are not unique to laboratory rats but can be observed also in humans. Translational studies in heroin and cocaine co-abusers have shown that addicts prefer the home setting for heroin use and non-home settings for cocaine use, regardless of the route of drug taking (Caprioli et al. 2009; Badiani and Spagnolo 2013).

The mechanisms responsible for the substance-specific influence of setting on drug taking are not known. It has been proposed that the setting of drug taking might provide an ecological background against which the central and peripheral effects of drugs are appraised (Badiani 2013). In the presence of a “mismatch” between the setting and the internal state of the organism, the rewarding effects of the drug would be reduced. According to this hypothesis, the central and peripheral (sympathomimetic) arousal produced by cocaine would be appraised as appropriate to arousing non-home settings but not to the home environment. In contrast, the central and peripheral (parasympathomimetic) sedation produced by heroin would be consistent with the safety of the home environment but not to potentially challenging non-home settings.

In the present study, we used the ultrasonic vocalizations (USVs) emitted by rats self-administering heroin and cocaine, as an index of the affective state of the rats. Indeed, 50-kHz USVs are emitted in response to rewarding stimuli, such as intraspecific play (Knutson et al. 1998), hetero-specific play/tickling (Burgdorf and Panksepp 2001; Mällo et al. 2007; Panksepp and Burgdorf 2000, 2003; Schwarting et al. 2007; Wöhr et al. 2009), sex (McGinnis and Vakulenko 2003; White et al. 1990; Bialy et al. 2000), food (Burgdorf et al. 2000), electrical stimulation of the medial forebrain bundle (Burgdorf et al. 2000), and exposure to addictive drugs (Ahrens et al. 2009; Knutson et al. 1999; Natusch and Schwarting 2010; Wintink and Brudzynski 2001; Wright et al. 2010; Barker et al. 2010; Maier et al. 2010). In contrast, 22-kHz USVs are emitted in association with exposure to aversive stimuli, such as electrical footshock (Lee et al. 2001; Koo et al. 2004) and predators (Blanchard et al. 1991, 1992), drug withdrawal (Covington and Miczek 2003; Mutschler and Miczek 1998; Vivian and Miczek 1991), defensive or submissive postures during intraspecific aggression (Lore et al. 1976; Portavella et al. 1993; Thomas et al. 1983), and chronic pain (Calvino et al. 1996). Thus, it has been proposed that 22-kHz USVs reflect negative internal affective states of the lab rat, whereas 50-kHz USVs reflect positive affective states (see Barker 2010).

## Materials and methods

### Animals

A total of 32 male Sprague–Dawley (Harlan Laboratories) rats weighting 250–280 g at the beginning of the experiment were used. Four rats were excluded from the analysis because they did not reach the SA criterion (at least two infusions per session during the last six sessions of SA for at least one substance). One rat died during the experiment. The rats were housed and tested in the same dedicated temperature- and humidity-controlled rooms (21 ± 1 °C; 70 %), with free access (except during the test sessions) to food and water under a reverse 14-h dark/10-h light cycle (lights off at 7:00 a.m.). The rats were gently handled twice a week for 2 weeks before undergoing catheterization surgery.

### Catheter surgery

On the day of surgery, the rats received an i.p. injection of 2.33 mg of xylazine hydrochloride (Rompun®, Bayer HealthCare) and 0.56 ml/kg of Zoletil 100® (Virbac, Carros, France), containing tiletamine (50 mg/ml) and zolazepam (50 mg/ml). The catheter consisted of two pieces of silicone tubing of 10.5 cm (0.37-mm inner diameter, 0.94-mm outer diameter) sheathed and held together at 3.4 cm from their proximal end by 5-mm-long heat-shrink tubing. By using standard surgical procedures, this double-lumen catheter was inserted into the right jugular vein and secured to the surrounding soft tissues with silk thread. Catheter distal ends were externalized through a small incision at the nape of the neck and connected to two L-shaped 22-gauge cannulae, which were secured to rat’s skull using dental cement and stainless steel screws. After surgery, the rats were given 15 mg i.v. enrofloxacin (Baytril®, KVP Pharma + Veterinär Produkte Gmbh, Kiel, Germany). Catheters were flushed daily with 0.1 ml of a sterile saline solution containing 0.4 mg of enrofloxacin and 25 IU heparin (Marvecs Services, Agrate Brianza, Italy).

### Self-administration procedures

After the surgery, the rats were assigned to the Resident or Non-Resident group. Resident rats were housed in the SA chamber throughout the experiment, whereas Non-Residents were housed in standard polycarbonate cages and were transferred to SA chambers only for the daily self-administration session (for more detail on apparatus and housing procedures, see Caprioli et al. 2007a). The catheters of Resident rats were connected to the infusion lines 3 h before the start of the SA session. The rats were trained to self-administer cocaine (400 μg/kg per infusion) and heroin (25 μg/kg per infusion) on alternate days for 14 consecutive daily sessions (3 h per session). These drug doses (dissolved in sterile saline) were selected on the basis of previous studies (Caprioli et al. 2007a, b; 2008; 2009; Celentano et al. 2009). For half of the rats, the starting drug was heroin and for the other half it was cocaine, and each drug was paired with one of the two levers in a between-subject counterbalanced manner. At the beginning of each session, the appropriate lever was extended and the relative cue light was switched on. Completion of the task on the lever resulted in the delivery of the infusion (40 μl) over a 3-s period and in the retraction of the lever and the switching off of the cue light for a 40-s timeout period. The rats that did not spontaneously self-administer at least one infusion within the first 5 min of the session were placed with their forepaws on the lever to prime an infusion. This was repeated at times 60 and 120 min for rats that did not self-administer at least one infusion in time periods 5–60 and 60–120 min. These priming infusions were not included in data analysis. The schedule requirement to obtain an infusion was progressively increased from fixed ratio 1 (FR1) to FR5 according to the following schedule: FR1 on sessions 1–6, FR2 on sessions 7–8, and FR5 on sessions 9–14. The lever alternation continued on sessions 15–16, but upon completion of the task (FR5), the rats received a saline injection.

### Ultrasonic vocalizations

**Fig. 1:**
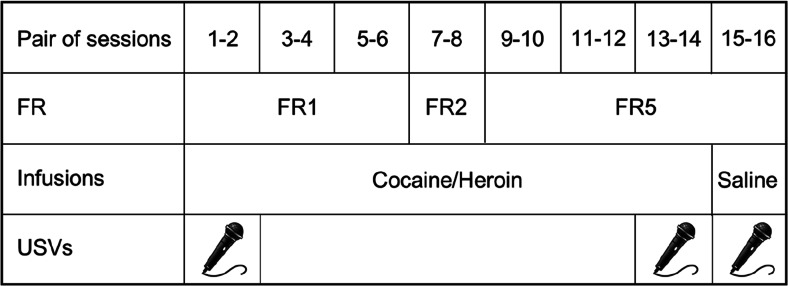
Outline of the experiment.  The microphones indicate the sessions during which USVs were recorded. Please note that during saline self-administration, the alternation between cocaine- and heroin-paired cues and lever position was maintained

**Fig. 2:**
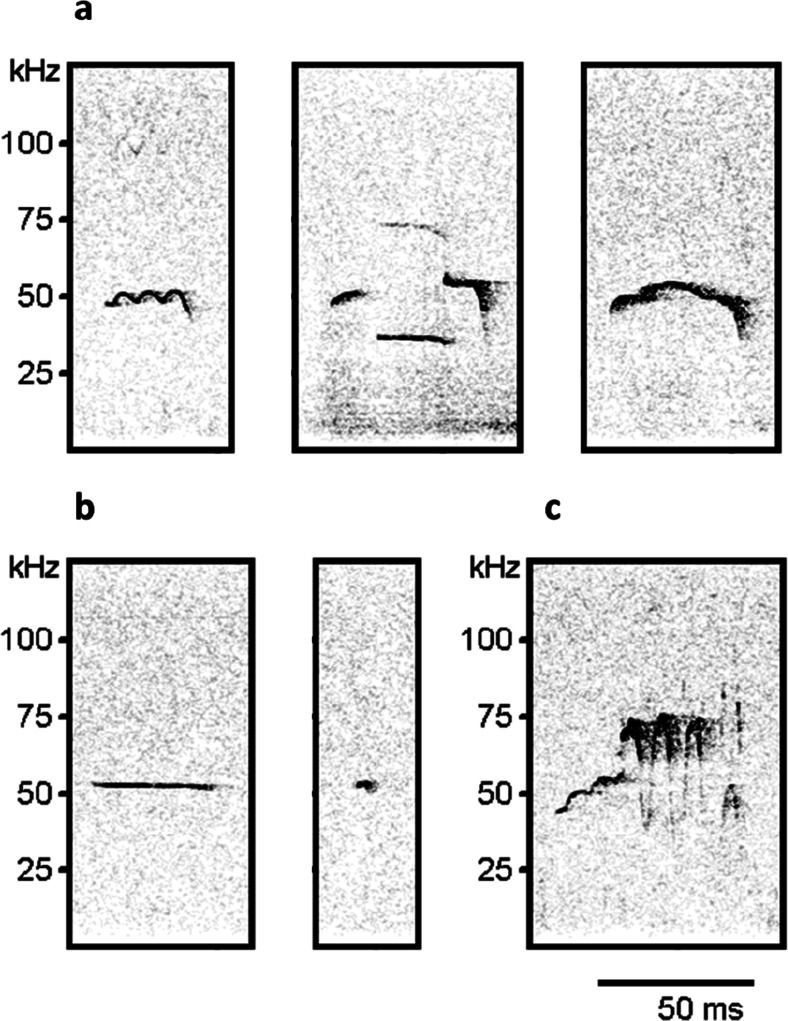
Representative spectrograms for three main categories of 50-kHz USVs. **a** Frequency-modulated calls are defined as vocalizations continuously or discretely modulated, with a mean slope >0.2 kHz/ms or with one or more pitch-jumps in them, which is an instantaneous change in frequency. **b** Fixed frequency calls have no modulation, with a mean slope of less than 0.2 kHz/ms. **c** Trills vocalizations are characterized by a rapid, massive frequency excursion, either alone or in combination with other calls

Ultrasonic vocalizations were recorded at baseline condition (3 min in a clean polycarbonate cage), during the period 0–30 min of the first two and the last two SA sessions, and again during the two sessions of saline SA (see Fig. 1). Avisoft UltraSoundGate condenser microphones capsule CM16 and Avisoft Recorder software (Version 3.2) were used. The recording settings included sampling rate at 250-kHz, 16-bit format. The recordings were processed using Avisoft SASLab Pro (Version 4.40) and a fast Fourier transformation (FFT). Spectrograms were generated with an FFT length of 1024 points and a time window overlap of 75 % (100 % Frame, Hamming window). The spectrogram was produced at a frequency resolution of 488 Hz and a time resolution of 1 ms. A lower cutoff frequency of 15 kHz was used to reduce background noise. Distinct calls were identified on the basis of USV-free intervals ≥50 ms. Each call was visually and acoustically identified by a trained observer and assigned to 1 of 15 categories (Wright et al. 2010), which were then further classified into three main categories, based on previous literature (Brudzynski 2015): (1) “frequency-modulated” (FM) calls, characterized by a continuous or discrete frequency modulation (≥0.2 kHz/s), in either one or two or more directions; (2) “fixed frequency” calls, which were substantially flat USVs (mean change in frequency ≤0.2 kHz/s; (3) “trills” defined as rapid, massive frequency oscillations (including their combinations with vocalizations from other categories); and (4) 22-kHz calls. Representative spectrograms for these USV categories are reported in Fig. 2.

### Statistics

Self-administration data were analyzed with a three-way mixed ANOVA with repeated measures on the factor drug (cocaine vs. heroin) and the factor session, and with setting as a between-subject factor. When the sphericity assumption was violated, Greenhouse–Geisser correction was adopted. Post hoc *t* tests for paired (when confronting lever pressing behavior on pairs of session from the same group) or unpaired samples (when confronting lever pressing behavior on sessions for the same substance between groups) were used to assess differences between sessions. Ultrasonic vocalization data were analyzed, due to high individual variability and lack of normal distribution, using Wilcoxon signed-rank tests for each subcategory. A drug preference score was obtained by calculating the ratio of USV emitted in response to cocaine versus heroin for each animal, after logarithmic normalization: log_10_[(USV_coc_ + 1)/(USV_hero_ + 1)]. Two-way mixed ANOVA was run on these data followed by post hoc one-tailed *t* tests, as the direction of change was clearly predicted on the basis of the working hypothesis. Data from three rats (two Non-Residents, one Resident) during the first session were lost due to hardware malfunctioning. Analysis was conducted using IBM SPSS 21.0 statistical software.

Separate analyses were conducted on the 50-kHz calls emitted immediately before (10 s) and immediately after the first ten infusions for sessions 13–14 and 15–16. Given the design of our study, there was large between- and within-subject variability in the number of cocaine, heroin, and saline infusions, as well as in their temporal distribution. Therefore, these data were analyzed using descriptive statistics only (see Figs. 7 and 8), as they were not suitable to inferential statistics.

## Results

### Self-administration

**Fig. 3:**
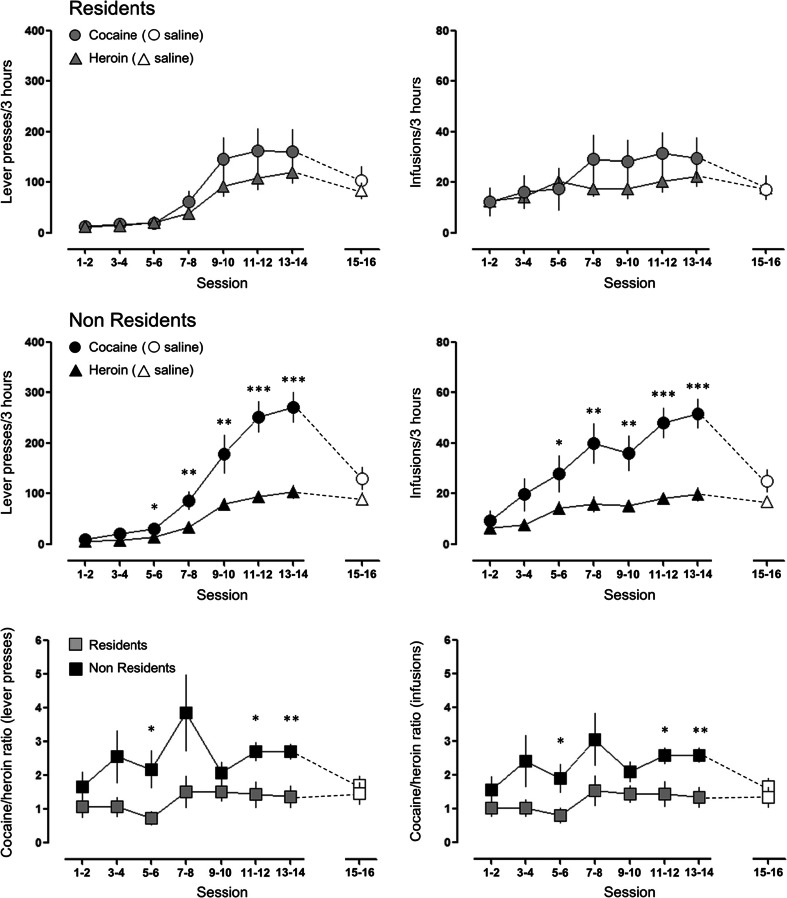
Self-administration of cocaine, heroin, and saline in Resident versus Non-Resident rats. *Left panels* illustrate the number of lever presses (means ± SEM) and *right panels* the number of infusion (means ± SEM) for each pair of sessions (see “Materials and methods” section). *, **, and *** indicate significant differences (*p* ≤ 0.05, *p* ≤ 0.01, and *p* ≤ 0.001, respectively) between cocaine and heroin

As illustrated in Fig. 3, cocaine SA and heroin SA were affected in a different manner by the setting. A three-way mixed ANOVA for repeated measures indicated significant main effects of session [F_6,150_ = 56.700; *p* < 0.001] and drug [F_1,25_ = 23.754; *p* < 0.001], and drug × setting [F_1,25_ = 5.818; *p* = 0.024], session × drug [F_6,15_ = 16.176; *p* < 0.001], and drug × session × setting [F_6,150_ = 4.290; *p* = 0.012] interactions. Virtually identical results were obtained analyzing earned infusions, with significant main effects of session [F_6,150_ = 14.328; *p* < 0.001] and drug [F_1,25_ = 17.347; *p* < 0.001], and drug × setting [F_1,25_ = 5.230; *p* = 0.031], and session × drug [F_6,15_ = 6.786; *p* < 0.001] interactions. No group differences were found for the saline SA sessions. The bottom panel of Fig. 2 illustrates the ratio of cocaine to heroin infusions. Two-way mixed ANOVA shown a main effect of setting (F_1,25_ = 10.294; *p* = 0.004).

### Ultrasonic vocalizations

**Fig. 4:**
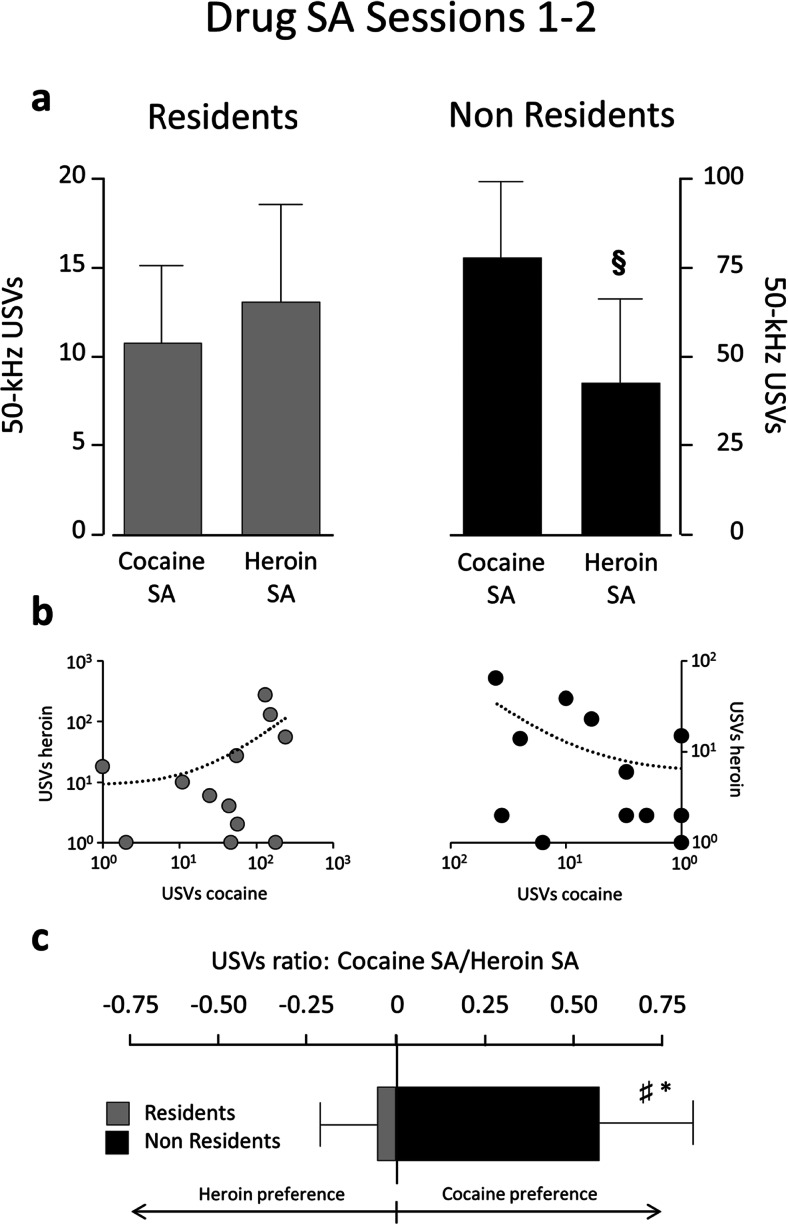
Fifty-kilohertz USVs emitted by Non-Resident and Resident rats during the first 30 min of drug SA for sessions 1–2. **a** Total number of calls (means ± SEM) for cocaine versus heroin SA. **b** Scatterplots of calls emitted during cocaine versus heroin SA. Each dot represents a single rat. **c** Preference score is calculated as the ratio of log-transformed calls emitted during cocaine versus heroin SA. **§** indicates significant difference (*p* ≤ 0.05) between cocaine and heroin. * indicates significant difference (*p* ≤ 0.05) between Residents and Non-Residents. # indicates significant difference (*p* ≤ 0.05) from 0

**Fig. 5:**
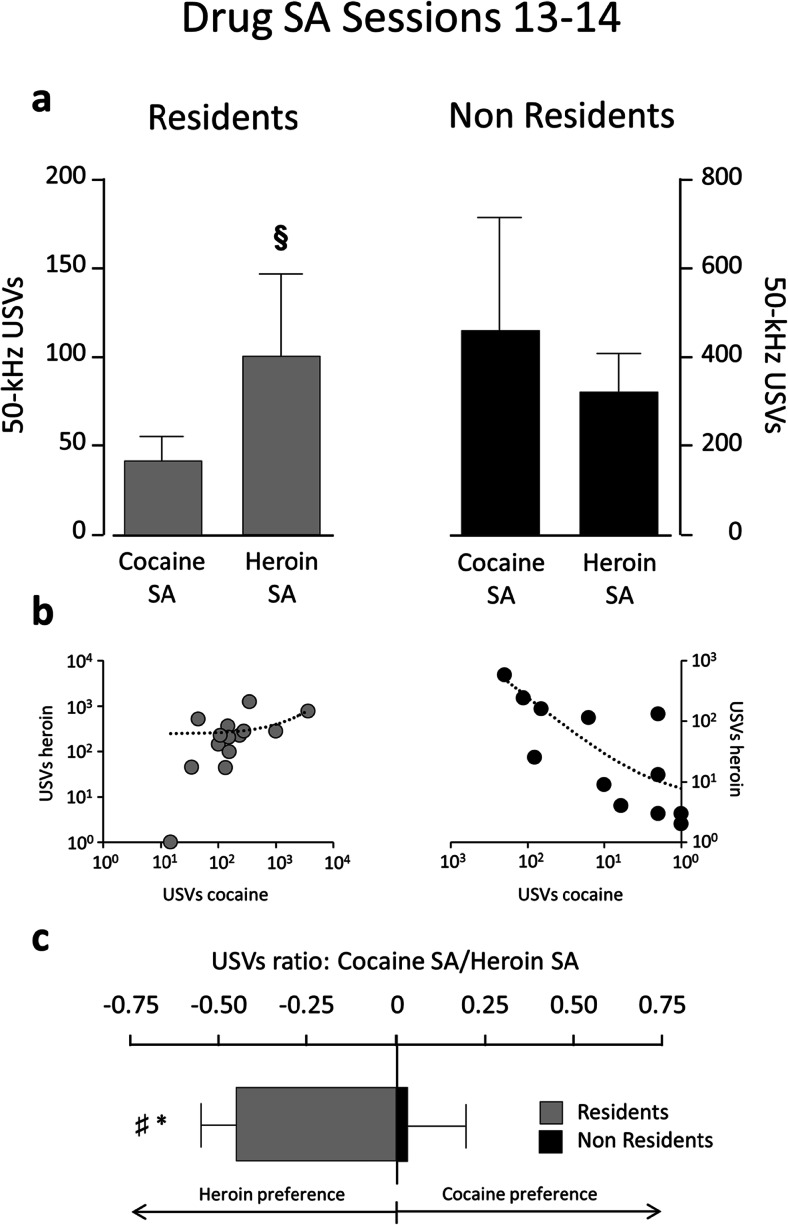
Fifty-kilohertz USVs emitted by Non-Resident and Resident rats during the first 30 min of drug SA for sessions 13–14. **a** Total number of calls (means ± SEM) for cocaine versus heroin SA. **b** Scatterplots of calls emitted during cocaine versus heroin SA. Each dot represents a single rat. **c** Preference score is calculated as the ratio of log-transformed calls emitted during cocaine versus heroin SA. **§** indicates significant difference (*p* < 0.05) between cocaine and heroin. * indicates significant difference (*p* ≤ 0.05) between Residents and Non-Residents. # indicates significant difference (*p* ≤ 0.05) from 0

Figures 4 and 5 illustrate the number of 50-kHz USVs emitted during the first 30 min of drug SA for sessions 1–2 and 13–14. Overall, Non-Resident rats produced more USVs than Resident rats. However, Non-Resident rats emitted more USVs in response to cocaine than in response to heroin, especially during sessions 1–2 (Fig. 4), when they produced about twice as many USVs for cocaine as for heroin (*p* = 0.039; *r* = 0.42). In contrast, Resident rats emitted more USVs in response to heroin than to cocaine, especially during sessions 13–14 (Fig. 5), when they produced about three times as many USVs for heroin as for cocaine (*p* = 0.044; *r* = 0.39). Figures 4 and 5 also illustrate the log-normalized ratios of cocaine-induced over heroin-induced USVs, further indicating that Non-Resident rats vocalize more in response to cocaine than to heroin during the early SA sessions, whereas Resident rats vocalize more in response to heroin than to cocaine during the last SA sessions. Bottom panels in Figs. 4 and 5 show the drug preference score (calculated as described in the “Materials and methods” section) for Resident and Non-Resident rats. A two-way mixed ANOVA for repeated measures conducted on these data indicated a main effect of session (F_1,22_ = 5.256; *p* = 0.032) and of setting (F_2,44_ = 4.006; *p* = 0.025). Post hoc *t* tests revealed a significant difference between Residents and Non-Residents at both early (t_24_ = −1.732; *p* = 0.048) and late training (t_25_ = −1.790; *p* = 0.043), but not for saline self-administration (t_25_ = −0.597; *p* = 0.556). Furthermore, the average of the scores of Non-Residents is significantly different from 0 for early training (t_13_ = 2.081; *p* = 0.031), whereas Resident rats’ scores differ from zero for late training (t_12_ = −2.267; *p* = 0.022).

**Fig. 6:**
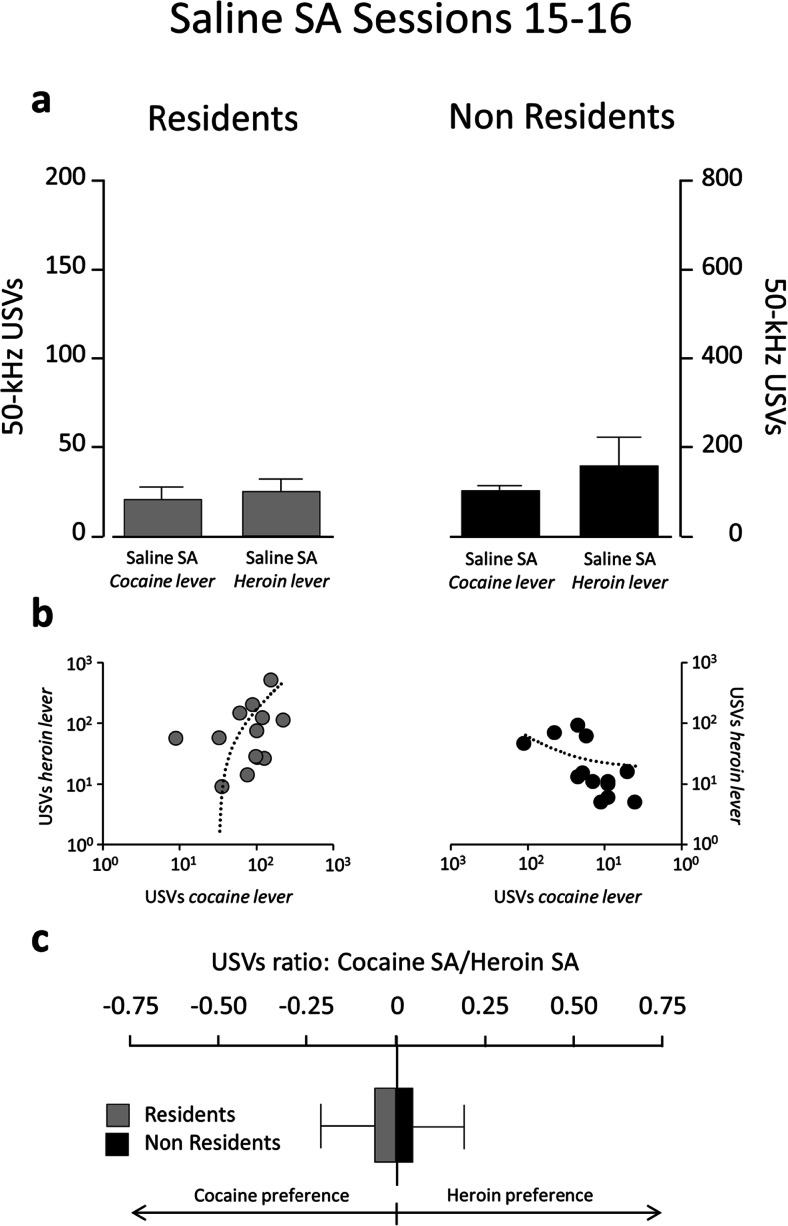
Fifty-kilohertz USVs emitted by Non-Resident and Resident rats during the first 30 min of saline SA for sessions 15–16. **a** Total number of calls (means ± SEM) when exposed to cocaine-paired versus heroin-paired cues. **b** Scatterplots of calls emitted when exposed to cocaine-paired versus heroin-paired cues. **c** Preference score, for cocaine versus heroin, is calculated as the ratio of log-transformed calls emitted when exposed to cocaine-paired versus heroin-paired cues

**Fig. 7:**
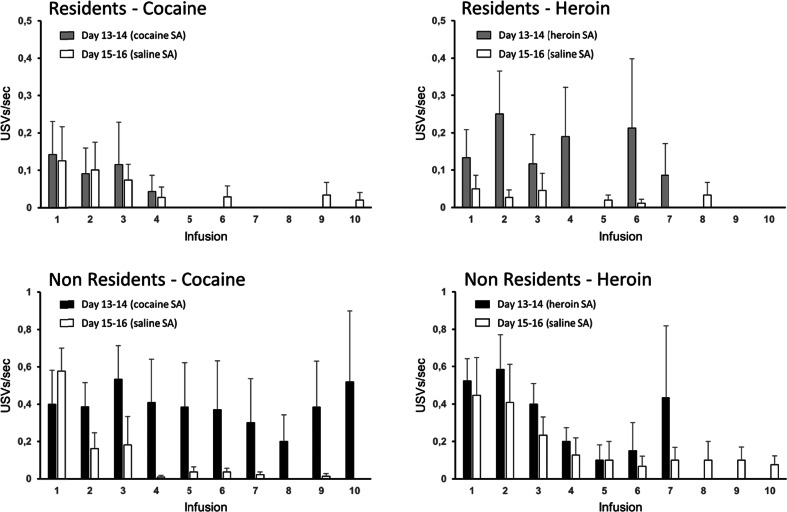
Pre-infusion calls. Rate of 50-kHz USVs (means ± SEM) in the 10 s before each of the ten first infusions on sessions 13–14 (heroin or cocaine infusions) versus sessions 15–16 (saline infusions). Due to great individual variability in number and timing of earned infusions, only descriptive statistics are displayed for this dataset (see “Materials and methods” section)

**Fig. 8:**
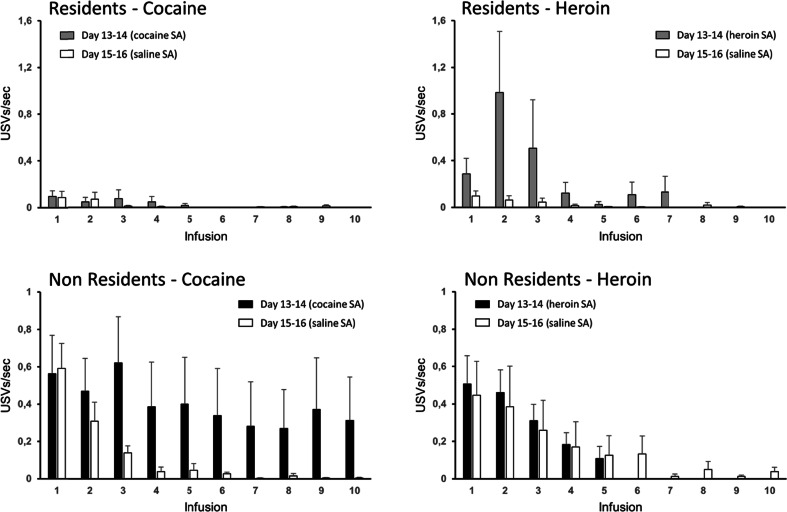
Post-infusion calls. Rate of 50-kHz USVs (means ± SEM) during the 40 s after each of the ten first infusions on sessions 13–14 (heroin or cocaine infusions) versus sessions 15–16 (saline infusions). Due to great individual variability in number and timing of earned infusions, only descriptive statistics are displayed for this dataset (see “Materials and methods” section)

The differential modulatory influence of setting on cocaine- versus heroin-induced USVs was critically dependant on the actual infusion of heroin or cocaine because it was not observable when the rats were exposed to the conditioned stimuli associated to drug infusion, as during saline SA on sessions 15–16 (Fig. 6). This phenomenon is even more evident when the calls emitted immediately before or after each infusion are considered. Figure 7 compares the frequency of preinfusion calls (10 s before infusion) for the first ten infusions of cocaine or heroin, on sessions 13–14, to that for the first ten infusions of saline, on sessions 15–16. Figure 8 illustrates a similar comparison for the calls emitted in the 40 s after each infusion. In the Resident group, the rats vocalized much more before and after heroin infusion than after saline infusion, whereas the call frequency for cocaine was similar to that for saline. In contrast, Non-Resident rats vocalized more before and after cocaine infusion than after saline infusion, whereas the call frequency for heroin was similar to that for saline.

There was no significant correlation between the number of heroin or cocaine infusions and the number of calls in any session for either the Resident or the Non-Resident rats (all *p* values ≥0.2; data not shown).

Table 1 illustrates the number of 50-kHz USVs for each category. In Non-Resident rats, cocaine elicited more frequency-modulated calls relative to heroin during sessions 1–2 (*p* = 0.05, *r* = 0.40). In contrast, Resident rats emitted more frequency-modulated (*p* = 0.032, *r* = 0.42) and trills (*p* = 0.043, *r* = 0.39) USVs in response to heroin relative to cocaine during sessions 13–14.

**Table 1:** Number (means ± SEM) of trills, frequency-modulated (FM) calls, and fixed frequency (FF) 50-kHz USVs (see text), emitted by Non-Resident and Resident rats during the first 30 min of drug SA (sessions 1–2 and 13–14) and saline SA (sessions 15–16)

		Residents		Non-Residents		All	
Session	USV category	Cocaine lever	Heroin lever	Cocaine lever	Heroin lever	Cocaine lever	Heroin lever
1–2 drug SA	Trills	0.58 ± 0.49	1.33 ± 1.33	8.33 ± 3.70	2.92 ± 1.59	4.18 ± 1.78	2.125 ± 1.03
	FM	4.92 ± 2.22	3.33 ± 1.70	* 45.17 ± 14.13	24.25 ± 13.71	* 25.4 ± 7.66	13.79 ± 7.10
	FF	5.58 ± 1.87	8.67 ± 3.63	24.00 ± 5.93	16.00 ± 8.38	15.96 ± 3.90	12.33 ± 4.53
	Total	10.23 ± 3.91	13.33 ± 5.70	* 78.36 ± 20.65	43.17 ± 23.57	45.55 ± 12.59	28.25 ± 12.26
13–14 drug SA	Trills	1.92 ± 0.81	* 16.62 ± 9.00	# 58.71 ± 29.70	68.00 ± 32.83	31.37 ± 16.12	43.26 ± 17.97
	FM	21.08 ± 10.28	*,# 53.31 ± 23.65	# 299.29 ± 186.02	## 182.71 ± 48.55	# 165.33 ± 98.68	## 120.41 ± 29.94
	FF	14.69 ± 6.13	28.31 ± 13.76	100.86 ± 42.73	66.07 ± 14.71	59.37 ± 23.52	47.89 ± 10.58
	Total	37.69 ± 16.94	*,# 98.23 ± 45.61	# 458.86 ± 255.88	# 316.79 ± 90.55	## 256.07 ± 136.90	## 211.55 ± 55.20
15–16 saline SA	Trills	1.38 ± 0.94	3.23 ± 2.60	14.43 ± 4.92	42.43 ± 20.02	8.15 ± 2.84	23.55 ± 10.96
	FM	7.92 ± 2.97	6.77 ± 3.02	44.14 ± 7.58	69.64 ± 28.73	26.70 ± 5.43	39.37 ± 15.94
	FF	9.62 ± 4.37	13.85 ± 5.09	38.57 ± 7.32	58.29 ± 28.35	24.63 ± 5.12	36.89 ± 15.27
	Total	18.92 ± 7.99	23.85 ± 8.16	97.14 ± 15.78	170.36 ± 75.91	59.48 ± 11.73	99.81 ± 41.41

*Significant differences (*p* ≤ 0.05) between heroin- and cocaine-induced calls; ^#^, ^##^significantly more (*p* ≤ 0.05 and *p* ≤ 0.01, respectively) drug-induced calls on sessions 13–14 relative to the corresponding saline session (sessions 13–14)

The rats emitted very few 22-kHz calls (about 1 % of all recorded calls). The majority of these 22-kHz calls (181 out of 200) were emitted by a single Non-Resident rat on the first session of heroin SA.

## Discussion

We report here three main findings. First, we found that the positive affective state (as indicated by 50-kHz USVs) induced by cocaine versus heroin SA is modulated in a substance-specific manner by the setting of drug taking. On the basis of previous studies (Caprioli et al. 2007a, b; 2008; 2009), we hypothesized that heroin is more rewarding than cocaine when self-administered in a familiar home environment, whereas cocaine is more rewarding when self-administered outside the home. Overall, the findings reported here are in agreement with this hypothesis.

Second, in agreement with previous reports (Barker et al. 2010; Browning et al. 2011; Ma et al. 2010; Maier et al. 2012; Reno et al. 2013), we found that cocaine SA facilitates the emission of 50-kHz USVs and that this phenomenon is temporally related to drug infusion, as indicated by the fact that the call frequency was higher in the periods immediately before and after the infusions relative to the rest of the session.

Third, we report here for the first time that heroin increases 50-kHz USVs and that, as for cocaine, this effect is temporally linked to drug infusion. To the best our knowledge, no previous study has examined the emission of 50-kHz USVs in rats self-administering heroin, or even morphine (which, in any case, has a pharmacological profile distinct from that of heroin; e.g., Antonilli et al. 2005).

We have previously reported that rats tend to self-administer more cocaine when the setting of drug taking is distinct from the home environment (Non-Resident rats) relative to when the SA chamber is also the home environment (Resident rats) (Caprioli et al. 2007a). In contrast, Resident rats tend to self-administer more heroin than Non-Resident rats (Caprioli et al. 2008). We also conducted experiments in which rats were trained, as in the present study, to self-administer cocaine and heroin (at the same dosages used here) on alternate days (Caprioli et al. 2009; Celentano et al. 2009; Montanari et al. 2015). Under such conditions, Non-Resident rats took much more cocaine than Resident rats whereas the two groups self-administered more or less the same amount of heroin, suggesting that the two drugs affected the intake of one another. Virtually identical results were reported here (see Fig. 2). We have previously discussed in detail the possible reasons for the differential reinforcing effects of cocaine and heroin as a function of setting (Caprioli et al. 2007b; Badiani 2013; Badiani and Spagnolo 2013). For example, although the relationship between the reinforcing and the discriminative effects of addictive drugs is a controversial issue (e.g., Gossop 2001), it is interesting to notice that the setting can affect in opposite directions cocaine and heroin discrimination (Paolone et al. 2004; Caprioli et al. 2007b), much in the same way it affects the self-administration of these two drugs. Thus, it is possible that when a drug is more easily discriminated, it also becomes more easily reinforcing. Another possibility is that the differences in the reinforcing effects of cocaine and heroin as a function of setting depend on differences in the hedonic properties of the two drugs. Heroin might be more reinforcing at home than outside the home because it induces a more positive affective state in the former setting than in the latter, and vice versa for cocaine. To investigate this hypothesis, we used USVs as an index of the emotional state of the rat. Research done in the past 25 years has shown that rats use USVs to communicate their emotional state to other conspecifics (for a review, see Brudzynski 2015). In particular, it has been shown that rewarding stimuli, including drug of abuse, can enhance the emission of 50-kHz USVs (Mutschler et al. 2001; Barker et al. 2010; Maier et al. 2010; Browning et al. 2011; Mahler et al. 2013). Thus, it has been proposed that these USVs may be used as an index of positive affective states in the rat (Knutson et al. 2002).

In the present study, we found major effects of setting and drug SA on the emission of USVs. First of all, Non-Resident rats emitted about ten times more 50-kHz USVs than Resident rats during both drug SA and saline SA. The most likely explanation for this finding is the heightened state of arousal produced by the transfer to a novel test environment (see Maier et al. 2010). Second, the number of USVs greatly increased over sessions in both Resident and Non-Resident rats. Sensitization of USV emission after repeated exposure to addictive drugs has been reported previously (Mu et al. 2009). Third, and most important, we found that the rate of USVs emitted during drug SA was modulated in a substance-specific manner by the setting. Specifically, the ratio of cocaine-induced to heroin-induced USVs was greater in Non-Resident than in Resident rats. The modulatory influence of setting on the emission USVs during cocaine and heroin SA was dependent on the presence of these drugs because it was no longer observable when the rats were shifted to saline SA.

The results summarized above are consistent with a hypothesis discussed in detail in previous papers (Badiani 2013; Badiani and Spagnolo 2013). Briefly, it was proposed that a drug is perceived as less rewarding when its peripheral and central effects are at odds with the setting of drug taking, that is, when there is a mismatch between setting and drug effects. The sympathomimetic, arousing, and activating effects of cocaine (or amphetamine), for example, would be experienced as unsuitable to a safe, non-challenging, domestic environment. In contrast, the drowsiness and sedation produced by heroin would be experienced as unsuitable to an exciting, novel environment. A similar line of reasoning would apply not only to psychostimulants and opiates. We have shown that Non-Resident rats take much more ketamine (which, like cocaine, has activating and sympathomimetic effects; Hancock and Stamford 1999) than Resident rats (De Luca and Badiani 2011), whereas Resident rats take more alcohol (which like heroin causes, at least initially, drowsiness and sedation; Morean and Corbin 2010) than Non-Resident rats (Testa et al. 2011).

The mismatch hypothesis would also account for an intriguing result of the present study, that is, for the fact that the modulatory effect of setting on the emission of USVs during drug SA changed in a substance-specific manner over time. Resident rats exhibited in fact no significant differences in the number of USVs emitted during heroin versus cocaine SA on sessions 1–2, whereas they emitted about three times more USVs during heroin SA relative to cocaine SA on sessions 13–14. It is possible that the first exposure to the testing procedures, including cue light presentation, lever extension, and drug infusion induced a certain degree of arousal, which waned with repeated testing. In contrast, Non-Resident rats emitted twice as many USVs during cocaine versus heroin SA on sessions 1–2, whereas there were no significant differences on sessions 13–14. It is possible that this was due to the repeated exposure of Non-Resident rats to the SA chamber, which might have blunted, but not erased, the relative novelty of the setting. However, it should be noted that when the analysis was limited to the USVs emitted immediately before or after drug infusion (Figs. 7 and 8), Non-Resident rats vocalized more before/after cocaine infusion than after saline infusion, whereas the number of peri-infusion calls for heroin was similar to that for saline.

While the mismatch hypothesis predicted greater rewarding effects of heroin in Resident versus Non-Resident rats and of cocaine in Non-Resident versus Resident rats, it did not necessarily predict greater aversive effects of heroin in Non-Resident versus Resident rats and of cocaine in Resident versus Non-Resident rats. In any case, under the testing conditions of the present study, the rats emitted very few 22-kHz USVs, which are thought to reflect aversive states (Blanchard et al. 1991, 1992; Calvino et al. 1996; Covington and Miczek 2003; Koo et al. 2004; Lee et al. 2001; Lore et al. 1976; Mutschler and Miczek 1998; Portavella et al. 1993; Thomas et al. 1983; Vivian and Miczek 1991). Interestingly, the majority of the very few 22-kHz calls recorded in our study (181 out of 200) were emitted by a single Non-Resident rat on the first session of heroin SA.

What are the neurobiological mechanisms responsible for the differential influence of settings on the emission of heroin- and cocaine-elicited calls? It has been previously shown that the intravenous administration of heroin and cocaine at doses identical to those used in present experiments differentially activate dorsal striatum neurons in Resident versus Non-Resident rats (Celentano et al. 2009). Given the role of the striatal complex in the production of 50-kHz USVs (Barker 2010), further studies are necessary to investigate whether this differential neuronal activation is at least in part responsible for the findings reported here.

In conclusion, the present study shows that the setting of drug administration modulates in a substance-specific manner not only the reinforcing and interoceptive effects of cocaine versus heroin (as shown in previous studies) but also the ability of these drugs to induce positive affective states, at least as reflected by 50-kHz USV. In particular, we have shown that a given setting of drug taking can modulate in opposite manner all aspects of heroin versus cocaine reward: intake (Caprioli et al. 2007a, 2008, 2009; Celentano et al. 2009), motivation (progressive ratio procedures; Caprioli et al. 2007a, 2008, 2009; Celentano et al. 2009), choice (Caprioli et al. 2009), drug discrimination (Paolone et al. 2004; Caprioli et al. 2007b), and affect (present study). It is important to notice that the setting does not influence all drug effects in the same way. We have previously shown that repeated administrations of heroin or morphine produce greater psychomotor sensitization in Non-Resident than in Resident rats (Badiani et al. 2000; Paolone et al. 2003, 2007), as previously reported for amphetamine and cocaine (Badiani et al. 1995a, b; Crombag et al. 1996; Browman et al. 1998). That is, psychomotor sensitization and rewarding effects can be modulated in opposite directions by the setting, and this opposite modulation has been observed to occur in parallel (e.g., Caprioli et al. 2008). Furthermore, some effects of drugs do not appear to be susceptible to the manipulation of setting investigated here. Tolerance to the analgesic effect of morphine, for example, develops in exactly the same way in Resident and Non-Resident rats (Paolone et al. 2003).

The effects of setting on cocaine versus heroin reward may explain the findings of studies conducted in human addicts, showing distinct setting preferences for cocaine versus heroin use (Caprioli et al. 2009; Badiani and Spagnolo 2013), and in rat models of drug relapse, showing differential vulnerability to cocaine versus primed reinstatement of drug seeking after a period of ext (Montanari et al. 2015). Taken together, these findings indicate the importance of taking into account the substance-specific aspects of drug use and misuse (Badiani et al. 2011; Badiani 2013).
